# The long road back to the start: a return to prothrombin complex concentrate

**DOI:** 10.1016/j.rpth.2026.103430

**Published:** 2026-03-26

**Authors:** Richard J. Buka, Raza Alikhan

**Affiliations:** 1 Department of Cardiovascular Sciences, College of Medical and Dental Sciences, University of Birmingham, Birmingham, UK; 2 West Midlands Adult Comprehensive Care Haemophilia and Thrombosis Centre, Department of Haematology, University Hospitals Birmingham National Health Service Foundation Trust, Birmingham, UK; 3 Department of Thrombosis & Haemostasis, Haematology Directorate, University Hospital of Wales, Cardiff and Vale University Health Board, Wales, UK; 4 Division of Population Medicine, Cardiff University School of Medicine, Cardiff, UK

Over the past decade, there has been a paradigm shift in anticoagulation use from vitamin K antagonists (VKAs) to direct oral anticoagulants (DOACs), particularly factor [F]Xa inhibitors (FXaIs). The risk of major bleeding, particularly intracerebral hemorrhage, with DOACs is significantly lower than with VKAs. Nevertheless, with the vast numbers of patients being anticoagulated globally for atrial fibrillation (AF), and, to a lesser extent, venous thromboembolism (VTE), there is a significant burden of morbidity and mortality associated with anticoagulation associated bleeding. The disappointing results with FXI inhibitors for AF and VTE mean that DOACs are here to stay, and the holy grail of preventing thrombosis, without bleeding, remains for now elusive.

Four-factor prothrombin concentrate (4F-PCC), which provides coagulation FII, FVII, FIX, and FX, is licensed for treatment of VKA-associated bleeding, but its widespread use in FXaI-associated bleeding is off-label. Andexanet, a catalytically inactive modified version of FXa, which acts as a decoy molecule, received accelerated approval from the US Food and Drug Administration (FDA) in 2019, but, in December 2025, the FDA announced that following the review of “postmarketing safety data on thromboembolic events, including serious and fatal outcomes,” it “considers the risks of the product to outweigh its benefit.” As a result, AstraZeneca withdrew andexanet from both manufacture and sale in the US market [1]. Andexanet alfa is therefore no longer available in the United States but is currently still available elsewhere in the world.

In this issue of *Research and Practice in Thrombosis and Haemostasis*, Shamiea et al. [2] report a timely retrospective analysis of 4F-PCC use for oral FXaI-associated intracerebral hemorrhage in a single center [2]. The study is particularly interesting because the authors applied the same inclusion and exclusion criteria as ANdexanet Novel EXA-Intracranial haemorrhage (ANNEXA-I), a randomized controlled trial that compared andexanet alfa with usual care for oral FXaI-associated acute intracranial hemorrhage (predominantly intracerebral) [3].

The cohort in the study by Shamiea et al. [2] comprised 52 patients, 87% of whom were taking apixaban. Baseline hematoma volume was modest (median, 5.5 mL; IQR, 2-21 mL) and lower than in ANNEXA-I. It is worth noting that although volume is a key predictor of outcomes, a vital modifier is the location of hemorrhage [4,5], neither of which are detailed in either study. As in ANNEXA-I, the primary end point was hemostatic efficacy, defined as a composite of hematoma expansion of <35% on follow-up imaging within 12 hours, a <7-point deterioration in National Institutes of Health Stroke Scale at 12 hours, and no requirement for rescue therapy within 3 to 12 hours of randomization.

A notable feature of the study is that it only included patients in whom hematoma expansion could be assessed, specifically those who underwent repeat imaging between 10 and 14 hours after the index scan. Hemostatic efficacy was achieved in 39 of 52 patients (75%), all of whom had hematoma expansion of <35%, with 1 thrombotic event (deep vein thrombosis) by 30 days. The 30-day all-cause mortality rate was 23%, which was comparable with the rates in ANNEXA-I. A repeat scan appears to reflect routine practice in their center, but it also introduces selection bias whereby patients who deteriorate early, proceed rapidly to palliation, undergo urgent neurosurgical intervention, or die may be systematically excluded. In addition to the small sample and variation, this may contribute to the better hemostatic efficacy than ANNEXA-I.

ANNEXA-I remains the highest-quality evidence in this arena and demonstrated a statistically significant improvement in hemostatic efficacy with andexanet alfa compared with usual care. This was driven primarily by a reduction in the number of patients with hematoma expansion of ≥35%. However, this apparent benefit was offset by a significantly increased risk of thromboembolic events, predominantly ischemic stroke, occurring within the first few days after treatment. This prompted the FDA’s unfavorable review of andexanet alfa and the subsequent decision by AstraZeneca to withdraw it from manufacture and sale in the United States.

Although andexanet alfa is still available worldwide, other professional groups and regulators may follow suit. In Europe, the European Medicines Agency previously reported a positive risk balance in April 2025 [6], but recent appraisal in January 2026 by the German Federal Joint Committee concluded that when andexanet was compared with 4F-PCC and best supportive care, there was an indication of less benefit in intracerebral hemorrhage and no added benefit in adults on apixaban or rivaroxaban with life-threatening or uncontrollable bleeding [7].

As such, the FDA decision now heralds a return to 4F-PCC as the default reversal strategy. However, 4F-PCC itself remains an unproven therapy. Its mechanism of action is to flood the circulation with clotting factors, providing substrate for thrombin generation and aiming to overcome FXa inhibition. While experimental models and laboratory studies suggest 4F-PCC can partially restore thrombin generation [8,9], this does not necessarily equate to a proven improvement in clinically meaningful outcomes. Furthermore, ANNEXA-I showed relatively poor restoration of anti-FXa activity in the usual care group, reinforcing the possibility that any benefit of 4F-PCC is modest, indirect, or highly dependent on context.

The safety profile of 4F-PCC is often assumed to be reassuring by comparison with andexanet alfa, but this should not breed complacency. In ANNEXA-I, thromboembolism occurred in 7% of patients in the usual care group by 30 days [10], substantially higher than expected in general hospitalized populations [11]. While this likely reflects the high baseline thrombotic risk in patients requiring anticoagulation, it would be surprising if administering supraphysiological concentrations of procoagulant factors were risk free. Thromboembolism rates were also approximately 7% in the Reversal of Active Dabigatran (REVERSE-AD) trial of idarucizumab for dabigatran reversal [12], suggesting that events of this magnitude may be driven by the clinical context and interruption of anticoagulation rather than the reversal strategy alone. However, the causal contribution of any reversal agent remains uncertain, given the absence of a robust untreated comparator and limited power to detect thrombotic safety signals in existing trials. 4F-PCC is also a pooled blood product, and while every effort to reduce risks is made, this risk is not negligible and may include unexpected, poorly appreciated long-term adverse effects [13].

A purely rational evidence-based approach would be to conduct a placebo-controlled randomized trial of 4F-PCC in FXaI-associated major hemorrhage. However, for many clinicians, randomization to placebo in this setting would feel deeply uncomfortable, and the feasibility of ethical approval and recruitment is questionable. When a medical practice evolves organically under the pressure to do something, it becomes increasingly difficult to generate the evidence required to know whether that something helps or harms. Meanwhile, the broader landscape offers limited relief. Novel bypassing agents are emerging, including VMX-C001, a modified FX molecule resistant to oral FXaIs [14]. This agent is now entering phase 3 trials in the periprocedural setting [15]. Although early-phase data are reassuring, population-level safety and efficacy are unproven, the cost is likely to be high, and evaluation in bleeding patients is a long way off.

In this context, the most urgent priority is not simply 4F-PCC vs the novel reversal agent, but to understand and rationalize how reversal decisions are currently made. Our recent United Kingdom–wide audit of reversal agent use demonstrated clear evidence of overuse of a therapy that should, in our view, be reserved for life-threatening bleeding [16]. Intracranial hemorrhage is particularly challenging because every bleed is, by definition, major [17]. However, it remains plausible that the majority of patients receiving reversal agents, even for intracranial hemorrhage, derive little or no benefit as they present many hours after symptom onset and after their last dose of anticoagulation [16].

Current practice is characterized by a patchwork of consensus-based protocols [18,19]. Most centers rely heavily on reported time since last anticoagulant dose to determine eligibility for reversal. Twenty-four hours are generally used as a cutoff, but in real-world bleeding patients, drug elimination is variable [20], and timing of last dose is an imprecise predictor of whether a patient is therapeutically anticoagulated.

Urgent drug-level testing is available in a minority of hospitals. However, evidence on implementation, clinical outcomes, and safety of level-guided reversal strategies remains scarce, and there are no widely adopted algorithms to integrate timing and drug levels into coherent decision making. Even where urgent levels are deliverable, the threshold for reversal is uncertain. The International Society of Thrombosis and Haemostasis recommendation of 50 ng/mL is grounded largely in laboratory measures (particularly thrombin generation) and limited periprocedural clinical data [21]; whether this threshold is optimal for bleeding patients is unknown. A final elephant in the room is dose. In both the study by Shamiea et al. [2] and ANNEXA-I, 4F-PCC dosing was not mandated and ranged from 25 to 50 units/kg. Our national audit similarly showed wide variability, compounded by different maximum doses for commonly used products (eg, Beriplex 5000 units vs Octaplex 3000 units) [16]; there remains uncertainty as to how much 4F-PCC should be given and how to tailor dosing to individual patient factors.

The ship of opportunity for a placebo-controlled 4F-PCC trial has long since sailed, but we can still aim to make meaningful improvements. Despite the lack of evidence to guide, limited standardization of approaches based on consensus could reduce variation, improve safety, and reduce errors. From an evidence-generation perspective, prospective pragmatic trials could randomize patients to different 4F-PCC doses, or to reversal at different drug-level thresholds, to evaluate safety and effectiveness. Rather than continued reliance on opinion, stepwise refinement of reversal practice is the most realistic path toward improving outcomes in the field of anticoagulation associated bleeding.
